# Family support in adults with epilepsy

**DOI:** 10.1055/s-0043-1777004

**Published:** 2023-11-30

**Authors:** Gloria M. A. S. Tedrus, Vania Aparecida Leandro-Merhi, Augusto Etchegaray, Yara Maria Randi

**Affiliations:** 1 Pontifícia Universidade Católica de Campinas, Escola de Ciências da Vida, Programa de Pós-Graduação em Ciências da Saúde, Campinas SP, Brazil.

**Keywords:** Epilepsy, Family Support, Quality of Life, Epilepsia, Apoio Familiar, Qualidade de Vida

## Abstract

**Background** The perception of family support in chronic disease can be relevant.

**Objective** To assess the perception of family support in adult patients with epilepsy (PWEs) and relate it to quality of life (QoL) and clinical aspects.

**Methods** Data from the Perceived Family Support Inventory (IPFS) of 130 PWEs were related to the clinical variables, QOLIE-31 scores, and the Neurological Disorders Depression Inventory for Epilepsy (NDDI-E) with statistical tests, with *p* < 0.05.

**Results** The mean age was 49.9 ± 17.2 years, and the duration of epilepsy was 20.8 ± 15.4 years. The presence of depression (scores ≥ 15) was associated with lower family support. Being married and non-occurrence of depression were the variables associated with a higher IPFS score (R = 0.2112), in the multiple linear regression.

**Conclusion** The perception of greater family support was associated with demographic aspects, the absence of depression, and better QoL. Family relationships may play an essential role in health adjustment behaviors and QoL in epilepsy.

## Resumo

**Antecedentes** A percepção do suporte familiar nas doenças crônicas pode ser relevante.

**Objetivo** Avaliar em pacientes adultos com epilepsia (PCE) a percepção do suporte familiar e relacioná-la com os aspectos clínicos e com a qualidade de vida (QV).

**Métodos** Os dados do Inventário de Percepção de Suporte Familiar (IPSF) de 130 PCE foram relacionados com as variáveis clínicas, os escores do QOLIE-31 e com o Neurological Disorders Depression Inventory for Epilepsy (NDDI-E), com testes estatísticos, com *p* < 0.05.

**Resultados** A idade média foi 49.9 ± 17.2 anos e o tempo de epilepsia foi de 20.8 ± 15.4 anos. Escores ≥ 15 no NDDI-E (presença de depressão) associaram-se a menor suporte familiar. Ser casado e não ter depressão são as variáveis associadas a maiores escores no IPSF (R = 0.2112), na regressão linear múltipla.

**Conclusão** A percepção de maior suporte familiar associou-se à aspectos demográficos, a ausência de depressão e a melhor QV. As relações familiares podem ter papel essencial nos comportamentos de ajustamento na saúde e na QV na epilepsia.

## INTRODUCTION

Epilepsy is a chronic neurological disorder and adult patients with epilepsy may have psychiatric and cognitive comorbidities, with frequent social repercussions and impaired quality of life (QoL). 1 It is known that patients with epilepsy have a worse perception of QoL when compared to the general population and people who suffer from other chronic illnesses. 2 3 In epilepsy, several clinical and psychosocial factors and the perception of social support are related to QoL. 4 5 6 7 The perception of family support in childhood epilepsy is well described in the literature. However, there is little literature that discusses the effects of family support in adult patients. Studies describe that individuals with chronic illnesses are more dissatisfied and perceive lower family support. 8 A better understanding of the perception of family support in epilepsy can increase knowledge of health-related QoL in epilepsy, and thus contribute to better guidance as a strategy to mitigate the stigma and social restrictions that adults with epilepsy may be subject to. The aim of this study was to assess the perception of family support in adult patients with epilepsy and relate these data to the perception of QoL and clinical variables.

## METHODS

This observational study consecutively included patients aged 18 years or older, treated at the clinical neurology outpatient clinic of the Hospital PUC-Campinas (Pontifícia Universidade Católica de Campinas), with a diagnosis of epilepsy according to the criteria of the International Classification of Epilepsy and the Epileptic Syndromes of the International League Against Epilepsy (ILAE). 9

Patients undergoing epilepsy surgery, with disabling chronic illnesses, with neurodegenerative illnesses, and with severe cognitive deficits were excluded from the study. All participants signed the informed consent form. The project was approved by the PUC-Campinas Human Research Ethics Committee, CAAE reference number: 13195619300005481; approval number: 3608734. All patients were interviewed individually in a hospital room, before their regular outpatient medical appointment.

### Procedures

The patients underwent the following assessments:

- Questionnaire for collecting demographic data (age, gender, educational level, and marital status) and clinical variables of epilepsy (age of onset, type, and frequency of epileptic seizures, and the number of antiseizure medications (ASMs) taken. Inventory of Perceived Family Support (IPFS), a questionnaire with 42 statements about understanding the perception of family support received by the patient. The inventory is evaluated by the total score and three dimensions: affectivity, adaptability, and autonomy. The higher the score, the better the individual's perception of the family support received. 10
- The Mini-Mental State Examination (MMSE) for cognitive tracking 11 12 ; the Quality of Life in Epilepsy Inventory (QOLIE-31) to assess the perception of QoL 13 14 ; and the Neurological Disorders Depression Inventory for Epilepsy (NDDI-E) to assess the presence of depressive symptoms (no = < 15; yes = ≥ 15). 15 16

### Statistical analysis

The IPFS scores (dimensions and total score) were related to clinical variables and MMSE, NDDI-E, and QOLIE-31 scores. To describe the characteristics of the sample, frequency tables were created for categorical variables with values of absolute frequency (n) and percentage (%), and for quantitative variables, descriptive measures were obtained (mean, standard deviation). To compare continuous measurements between the two groups, the Mann-Whitney test was applied. To assess the linear relationship between the variables, Spearman's correlation coefficient was used.

To assess the factors demographic (age, gender, marital status) and clinical variables (age of onset, length of epilepsy, type, and frequency of seizures, and number of ASMs taken) and scores on the QOLIE-31 and on the NDDI-E (presence of depression yes/no) related to the total score on the IPFS, multiple linear regression analysis was used, with a stepwise criterion for selecting variables. Variables were transformed into ranks due to the absence of normal distribution. The significance level adopted for the statistical tests was 5%.

## RESULTS

This study included 130 patients with epilepsy. The type of epileptic syndrome was genetic (generalized idiopathic) in nine (6.9%) cases, unknown etiology in 38 (29.2%) cases, and structural focal in 83 (63.8%) cases. The etiology of structural epilepsy was temporal lobe epilepsy with hippocampal sclerosis in 41 cases, vascular in 20 cases, traumatic brain injury in four cases, surgery for an intracranial tumor or vascular malformation in 10 cases, and other etiologies in eight cases. Clinical aspects and scores of the IPFS and of the QOLIE-31 are shown in Table 1 .

**Table 1:** Demographic aspects and clinical data of adult patients with epilepsy

Variables		(±or %)
Age (years)		49.9 ± 17.0
Educational level (years)		6.1 ± 4.0
Female sex		72 (55.4%)
Marital status – married		77 (59.2%)
Occupation – employed		59 (78.7%)
Inventory of perceived family support	Affectivity	32.1 ± 8.9
	Adaptability	20.9 ± 4.7
	Autonomy	13.2 ± 3.6
	Total score	66.1 ± 14.0
Age of onset (years)		29.0 ± 22.1
Length of epilepsy (years)		20.8 ± 15.4
Type of seizures: focal/generalized		108/22
Frequency of seizures: < once/year/≥once/year		67/63
Antiseizure medications: 1/≥2		85/45
Neurological disorders depression inventory for epilepsy	< 15	112
	≥ 15	17
QOLIE-31 (total score)		55.9 ± 28.4

Abbreviations: QOLIE-31, Quality of life in epilepsy inventory.

### IPFS: clinical aspects and QoL

There was no correlation between IPFS scores and age, educational level, age of onset, length of epilepsy, and MMSE scores. Patients with higher IPFS scores (total score and on the affectivity and adaptability dimensions) had lower scores on the NDDI-E. There was a positive and significant correlation between the scores on the QOLIE-31 and the total score and IPFS scores ( Table 2 ).

**Table 2:** Correlation values between IPFS scores and age, age of onset, and NDDI-E, MMSE, and QOLIE-31 scores

Variables		Total score		Family affectivity		Family adaptability		Family autonomy	
		r	*P*	r	*p*	R	*p*	r	*p*
Age		-0.04708	0.5992	-0.01025	0.9086	-0.03682	0.6799	0.02131	0.8113
Age of onset		-0.10027	0.2620	-0.05731	0.5205	-0.07101	0.4257	-0.05003	0.5750
NDDI-E (total score)		-0.21774	0.0139*	-0.18784	0.0345*	-0.22023	0.0125*	-0.08725	0.3274
MMSE		-0.05746	0.5211	-0.08025	0.3679	-0.04924	0.5810	0.01632	0.8550
QOLIE-31	Seizure worry	0.06193	0.4892	0.01638	0.8544	0.10258	0.2492	0.17377	0.0498*
	Overall QoL	0.11295	0.2061	0.08320	0.3505	0.19187	0.0300*	0.00191	0.9829
	Emotional well-being	0.15294	0.0861	0.17680	0.0459*	0.13926	0.1169	0.05167	0.5624
	Energy/ fatigue	0.21699	0.0143*	0.18523	0.0363*	0.27932	0.0014*	0.02987	0.7378
	Cognitive function	0.07302	0.4146	-0.01446	0.8713	0.17666	0.0461*	0.12970	0.1445
	Medication effects	0.07574	0.3974	0.03836	0.6672	0.23253	0.0083*	0.07361	0.4089
	Social function	0.08512	0.3414	-0.00795	0.9290	0.18885	0.0328*	0.13637	0.1248
	Total score	0.10408	0.2442	0.03995	0.6543	0.22987	0.0090*	0.12752	0.1515

Abbreviations: IPFS, Inventory of Perceived Family Support; MMSE, Mini-mental state examination; QOLIE-31, Quality of life in epilepsy inventory; NDDI-E, Neurological Disorders Depression Inventory for Epilepsy.

Notes: Spearman correlation; * *p* < 0.05.

When evaluating the perception of family support according to demographic and clinical aspects, it was observed that there was no significant difference in the IPFS according to gender, current frequency and type of epileptic seizure, number of ASMs taken, and type of epileptic syndrome. Married patients perceived more affection and greater family support (total score) when compared to those with other marital situations (single/widowed/separated). Patients with depression perceived less family support ( Table 3 ).

**Table 3:** Inventory of perceived family support according to demographic data and NDDI-E scores

Variables		Total score	Affectivity	Adaptability	Autonomy
Sex	Female (n = 72)	65.44 ± 14.57	31.28 ± 9.52	20.67 ± 4.73	13.56 ± 3.76
	Male (n = 58)	67.14 ± 13.51	33.21 ± 8.22	21.38 ± 4.82	12.82 ± 3.45
	p-value	0.4397	0.1796	0.2364	0.2057
Marital status	Married (n = 77)	68.29 ± 14.60	33.50 ± 9.19	21.43 ± 4.71	13.56 ± 3.58
	Others (53)	63.15 ± 12.82	30.15 ± 8.38	20.34 ± 4.82	12.77 ± 3.68
	p-value	0.0079*	0.0087*	0.1699	0.2710
Occupation	Employed (n = 59)	67.69 ± 12.25	32.90 ± 7.46	21.17 ± 4.20	13.63 ± 3.96
	Unemployed (n = 16)	62.53 ± 20.52	32.88 ± 13.19	19.07 ± 6.37	11.60 ± 4.12
	p-value	0.3966	0.7756	0.3795	0.1003
NDDI-E (total score)	< 15 (n = 112)	68.68 ± 12.37	33.37 ± 8.16	21.74 ± 4.02	13.61 ± 3.50
	≥ 15 (n = 17)	48.88 ± 13.29	22.63 ± 8.44	15.63 ± 6.14	10.63 ± 3.52
	p-value	<0.0001*	<0.0001*	<0.0001*	0.0006*

Abbreviation: NDDI-E, Neurological Disorders Depression Inventory for Epilepsy.

Notes: Mann-Whitney test; * *p* < 0.05.

Multiple linear regression analysis was applied in order to assess which demographic and clinical variables are related to the total IPFS score. It was observed that marital status and the presence of depression were the variables that are jointly associated with the total score on the IPFS. Married patients (R ^2^ 0.1756; *p* -value 0.0197) and those not classified as depressed (NDDI-E <15) (R ^2^ 0.0355; *p* -value < 0.0001) had higher values for the total score on the IPFS (better perception of family support).

## DISCUSSION

This study assessed the perception of family support and its relationship with clinical variables in individuals with chronic epilepsy. Despite the benefits of family involvement in chronic illnesses being well documented in the literature, there are still reports of difficulties in this relationship. The lack of social support and the presence of family dysfunction are the main problems observed in families of patients with severe chronic illnesses, particularly in those with mental disorders. 17 18 19

In different cultures, the psychosocial repercussions of epilepsy such as stigma, low self-esteem, social restrictions, and the negative impact on psychosocial well-being affect patients and families. 20 21 Family involvement in epilepsy, particularly in cases with epileptic seizures refractory to treatment, can mean challenges, conflicts, and sometimes overload, or even emotional distancing and denial, which can reflect on the bond between patients with epilepsy and family members.

Married patients with epilepsy perceive better family support than single/widowed/separated ones. A growing number of studies have demonstrated the importance of marital quality in dealing with the stress associated with the presence of chronic illnesses. Marital satisfaction is perceived as strong family and emotional support with positive repercussions in coping with illnesses. 22

Patients with epilepsy without depressive symptoms perceive greater family support, which may be related to the positive effects of greater engagement and family cohesion and emotional bonding with consequent reduction of stress and negative emotions. 22 23 It is known that families that are more cohesive and concerned about those with chronic illnesses tend to have early detection of warning signs of depressive symptoms, thus contributing to helping to reduce the severity of symptoms. 24

Better QoL was related to a greater perception of family support. It is known that greater family involvement is intrinsically associated with physical, emotional, and social health and with better QoL. 19 Better QoL perception in the well-being and energy/fatigue dimensions was associated with greater family affection, family cohesion, and emotional bonding.

This study has several limitations. Firstly, it is a cross-sectional study, and therefore, the causality of the relationships cannot be inferred. Another limitation is that the data were obtained from a single university hospital, with a small sample, so these findings should be interpreted with caution. However, the data obtained are relevant and unpublished, and, therefore, the use of data from this study is plausible.

In conclusion, family support was associated with demographic aspects, the absence of depression, and better QoL perception. Family relationships can play an essential role as providers of self-care and adjustment behaviors in the context of health and to maintain QoL in epilepsy.
